# Oral Squamous Cell Carcinoma With and Without a History of OPMDs: A Retrospective Study of Clinicopathological Characteristics

**DOI:** 10.3390/cancers18172776

**Published:** 2026-08-26

**Authors:** Gianluca Tenore, Ahmed Mohsen, Gian Marco Podda, Lucia Borghetti, Federica Rocchetti, Laura Sansotta, Andrea Battisti, Valentino Valentini, Antonella Polimeni, Umberto Romeo

**Affiliations:** Department of Oral and Maxillofacial Sciences (SOMF), Sapienza University of Rome, 00161 Rome, Italy; gianluca.tenore@uniroma1.it (G.T.); ahmed.mohsen@uniroma1.it (A.M.); lucia.borghetti@uniroma1.it (L.B.); federica.rocchetti@uniroma1.it (F.R.); sansotta.2041402@studenti.uniroma1.it (L.S.); andrea.battisti@uniroma1.it (A.B.); valentino.valentini@uniroma1.it (V.V.); antonella.polimeni@uniroma1.it (A.P.); umberto.romeo@uniroma1.it (U.R.)

**Keywords:** biopsy surveillance, de novo carcinoma, early diagnosis, oral cancer epidemiology, oral potentially malignant disorders, oral squamous cell carcinoma, risk factors

## Abstract

**Background:** Oral squamous cell carcinoma (OSCC) is a multifactorial malignancy traditionally associated with tobacco exposure, alcohol consumption, and human papillomavirus (HPV) infection. It may also arise through the malignant transformation of oral potentially malignant disorders (OPMDs). However, a substantial proportion of tumors arise without clinically detectable precursor lesions, suggesting heterogeneous carcinogenic pathways. This study aimed to compare the clinicopathological characteristics of OPMDs-associated and apparently de novo OSCC. **Methods:** A retrospective analysis was conducted on patients with histologically confirmed OSCC referred to the department between 2015 and 2025. Baseline patient characteristics, clinical, and histopathological diagnostic management data were collected from medical records, and patients were classified as the OSCC with OPMD group or the de novo OSCC group based on the presence or absence of a history of OPMDs. **Results:** Seventy-seven patients were included (mean age: 70.4 years; 53.2% males, 46.75% females). OPMDs were documented in 37.66% of cases, while 62.34% of tumors developed without documented precursor lesions. No significant difference was observed in histological grade distribution between the two groups (*p* = 0.206), although G1 tumors were more frequent in the OSCC with OPMD group (25.93%) than in the de novo OSCC group (9.30%). The de novo OSCC group showed a significantly higher prevalence of denture-related lesions. No significant differences were observed between the two considered groups in age, sex, tobacco or alcohol exposure, HPV status, systemic diseases, or oncological history. Patients with OPMDs underwent significantly more biopsies in total and biopsies per year. **Conclusions:** OSCC may develop through distinct pathogenic pathways, emphasizing the need for broader clinical vigilance beyond OPMD surveillance to improve early diagnosis.

## Simple Summary

A substantial proportion of oral squamous cell carcinomas develop without clinically detectable precursor lesions, making early diagnosis particularly challenging. Identifying differences between tumors arising from oral potentially malignant disorders and those developing apparently de novo may improve understanding of oral carcinogenesis and support more effective surveillance strategies. The aim of this retrospective study was to compare the clinical characteristics, risk factors, histopathological features, and diagnostic management of patients affected by oral squamous cell carcinoma with or without a history of precursor lesions. In a cohort of 77 patients, most tumors developed in the absence of recognized precursor lesions, while patients with oral potentially malignant disorders underwent more frequent biopsies and closer follow-up. These findings support the existence of heterogeneous carcinogenic pathways and highlight the need for careful clinical surveillance beyond patients with known precursor lesions.

## 1. Introduction

Oral squamous cell carcinoma (OSCC) represents a significant public health concern. It accounts for approximately 90% of diagnosed lip and oral cancers, which collectively rank as the 16th most common cancer worldwide, with an estimated 389,846 new cases and 188,438 deaths in 2022. In Italy, OSCC ranks as the 19th most frequently diagnosed cancer, with 4881 new cases and 1841 deaths recorded in 2022 [1].

The pathogenesis of OSCC remains incompletely understood and is widely recognized as multifactorial. Tobacco smoking and alcohol consumption, first highlighted by Franceschini and colleagues in 1990, have long been established as major risk factors for oral cancer [2]. Additional risk factors include human papillomavirus (HPV) infection—though its etiologic role appears limited in true oral cavity cancers compared to the oropharynx—as well as nutritional deficiencies, immune compromise, and hereditary susceptibility [3,4,5,6].

OSCC may arise de novo from clinically normal-appearing mucosa or develop from oral potentially malignant disorders (OPMDs), most commonly leukoplakia, erythro-plakia, and oral lichen planus or lichenoid lesions. The diagnosis of OPMDs relies on clinical evaluation and histopathological confirmation. In accordance with current clinical recommendations, any oral lesion persisting for more than two weeks after the elimination of potential local causative factors underwent biopsy for histopathological evaluation. The final diagnosis of each OPMDs was established through correlation of the histopathological findings with the clinical presentation [4]. Epithelial dysplasia may be absent or present, but when present, it is a key risk stratifier. Current evidence indicates that the likelihood of malignant transformation increases with dysplasia severity, being significantly higher in moderate–severe compared to mild dysplasia [7,8,9,10].

Not all OSCCs arise from clinically identifiable precursor lesions. The literature reports a significant portion of oral carcinomas arising de novo, without a previous diagnosis of OPMDs. Recent evidence suggests that carcinomas arising de novo may represent a distinct subset of OSCC and may differ from tumors associated with precursor lesions in terms of clinical characteristics and biological behavior and prognostic features [9,11].

This observation may be interpreted within the broader biological framework of field cancerization, which proposes that genetically altered epithelial areas can harbor malignant potential even in the absence of a clinically detectable precursor lesion [12,13,14].

The occurrence of OSCC in patients without a documented history of OPMDs (either clinically or histopathologically diagnosed) underscores the limitations of surveillance strategies focused exclusively on clinically evident pre-existing lesions. As a matter of fact, tumors arising de novo may become clinically apparent only after reaching a more advanced stage, underscoring the importance of identifying demographic, behavioral, and clinical risk factors associated with OSCC development [3,4].

In recent years, several epidemiological studies have also reported changes in the demographic and clinical profile of OSCC patients. Although the disease has traditionally been associated with aged individuals with long-term exposure to risk factors, as tobacco and alcohol, an increasing incidence has been observed among younger patients and among individuals without classic behavioral risk factors, particularly in cancers in-volving the oral tongue [15,16,17]. These findings suggest that oral carcinogenesis may involve heterogeneous pathways and highlight the need for continued investigation of patient risk profiles.

Understanding the clinical characteristics and risk factors of patients with OSCC remains essential for improving prevention strategies, identifying higher-risk individuals, and facilitating earlier diagnosis. A substantial proportion of OSCCs develop without a documented history of OPMDs, and available data on the frequency of de novo and OPMD-associated OSCC remain limited [11]. Further investigation of the clinical characteristics and diagnostic pathways of these patients may help address this gap, improve our understanding of these cases, and provide useful data for future analyses aimed at identifying factors that may contribute to earlier detection and prevention. The aim of the present study was to retrospectively analyze the risk profile of patients affected by oral squamous cell carcinoma, evaluating demographic characteristics, behavioral risk factors, clinical presentation, histopathological features, and diagnostic management, with particular attention to the presence or absence of history of oral potentially malignant disorders.

## 2. Materials and Methods

A retrospective study was conducted on patients referred to the oral surgery and oral medicine units of the Department of Oral and Maxillofacial Sciences, Sapienza University of Rome. The study protocol was approved by the institutional review board of the department (Prot. nr. 0000443, 27 February 2025), and all procedures were performed in accordance with the ethical principles of the Declaration of Helsinki and its subsequent amendments. Written informed consent was obtained from all participants.

### 2.1. Study Design and Data Collection

Medical records and institutional databases were retrospectively reviewed over a 10-year period (2015–2025). Patients aged ≥18 years with histologically confirmed OSCC were included in the study. Exclusion criteria consisted of incomplete clinical documentation, previous OSCC diagnosis established in external institutions without adequate supporting records, and age below 18 years. Given the retrospective design, no a priori sample size calculation was performed; all eligible patients meeting the inclusion criteria during the study period were included.

The collected variables included baseline patient characteristics, clinical, histopathological, and diagnostic management data. Baseline patient characteristics variables comprised age, sex, smoking and alcohol status, HPV history (based on anamnestic data recorded in the patients’ clinical records), previous solid or hematological malignancies, and concomitant systemic diseases. Clinical characteristics included tumor location, history of OPMDs, oral candidiasis, and denture-related lesions. Tumor sites were coded using ICD-O-3 topography codes (C01–C06) [18]. The anatomical location of each OSCC was recorded according to all oral subsites involved at diagnosis rather than exclusively to the primary site of origin. Consequently, a single tumor could be assigned to more than one anatomical category. Histopathological and diagnostic management data comprised the total number of biopsies, biopsy frequency (number of biopsies per year), and histological grade.

### 2.2. Statistical Analysis

Statistical analyses were performed using IBM SPSS Statistics for Windows, version 25.0 (IBM Corp., Armonk, NY, USA). Patients were stratified according to the presence or absence of a history of OPMDs with a minimum follow-up period of 6 months prior to OSCC diagnosis. The distribution of continuous variables was assessed for normality using the Shapiro–Wilk test. Comparative analyses between groups were performed using Pearson’s chi-square test or Fisher’s exact test for categorical variables, independent-samples *t*-test for normally distributed continuous variables, and Mann–Whitney U test for non-normally distributed variables. For categorical variables with more than two categories, overall comparisons were performed, and effect estimates were reported with 95% confidence intervals where appropriate. Multivariable binary logistic regression was performed using OPMDs history as the dependent variable and age, sex, smoking status, and histological grade as covariates, with adjusted odds ratios (aORs) and 95% confidence intervals reported. Cases with unspecified histological grade were excluded, and missing data were not imputed. Given the exploratory nature of the study, the potential for type I error due to multiple comparisons was considered. All tests were two-sided, and a *p*-value <0.05 was considered statistically significant.

## 3. Results

### 3.1. General Overview of the Study Population

A total of 77 patients with histologically confirmed diagnosis of OSCC were included in the study. The baseline patient characteristics, clinical, histopathological, and diagnostic management characteristics of the study population are summarized in Table 1.

The mean age of the cohort was 70.42 (±13.47) years (range 32–99 years), with most patients being older than 60 years (79.22%). Sex distribution was substantially balanced, with a slight predominance of males (53.25%) over females (46.75%).

Regarding behavioral risk factors, 25.97% of patients were current smokers, 27.27% were former smokers, and 46.75% had never smoked. Alcohol consumption was reported in 11.69% of cases, whereas 2.60% were former drinkers and the majority of patients (85.71%) denied alcohol use. HPV positivity was documented in 5.19% of the cohort.

Concomitant systemic diseases were highly prevalent, affecting 88.31% of patients. The most frequently reported conditions included arterial hypertension (58.82%), bone diseases (26.47%), cardiopathy (22.06%), diabetes mellitus (20.59%), and thyroid disorders (14.71%). Previous solid or hematological malignancies were identified in 19.48% of cases.

Overall, 37.66% of patients presented with at least one clinically documented OPMDs, whereas 62.34% had no documented precursor lesions. Among OPMDs, oral leukoplakia and oral lichenoid lesions represented the most frequent lesions (31.03% each), followed by oral lichen planus and erythroplakia (13.79% each). Proliferative oral leukoplakia was observed in 10.34% of OPMDs patients.

The most commonly involved anatomical sites of OPMDs were the buccal mucosa and gingiva (22.08% each), followed by the lateral border of the tongue (20.78%) and ventral tongue (19.48%).

Histopathological examination demonstrated a predominance of moderately differentiated carcinomas (G2, 55.84%), followed by poorly differentiated tumors (G3, 20.78%) and well-differentiated carcinomas (G1, 14.29%). Histological grade was unavailable in 9.09% of cases.

The tongue represented the most frequent anatomical site of OSCC (54.55%), particularly the lateral border of tongue tumors (66.67%). Other commonly involved sites included the gingiva (33.77%), buccal mucosa (24.68%), palate (16.88%), and floor of the mouth (6.49%).

Regarding diagnostic management, the mean total number of biopsies performed per patient was 2.1 (±2.0), with a mean of 1.5 (±1.0) biopsies per year.

### 3.2. Comparative Analysis Between Patients with and Without OPMDs

The comparative analysis of demographic, behavioral, clinical, and histopathological characteristics between patients with and without OPMDs history (Table 2) revealed substantial overlap in demographic and behavioral features. Mean age was comparable between groups (70.83 vs. 70.17 years), and no statistically significant differences emerged regarding sex distribution.

Similarly, no significant differences were observed for tobacco exposure, alcohol consumption, or HPV positivity. The distribution of current smokers, former smokers, and non-smokers was comparable between groups, as was alcohol consumption status. HPV positivity remained infrequent in both cohorts and showed no significant association (*p* = 0.6294). Likewise, concomitant systemic diseases and previous oncological history did not significantly differ between groups.

A statistically significant difference was observed regarding the diagnostic management patterns. Patients with OPMDs underwent a significantly higher total number of biopsies compared with patients without OPMDs (median 2 (IQR 1–3) vs. 1 (IQR 1–1.25); *p* = 0.0071; rank-biserial r = 0.32, 95% CI 0.09–0.54). Similarly, the number of biopsies performed per year was significantly higher in the OSCC with OPMD group (median 1 (IQR 1–2) vs. 1 (IQR 1–1); *p* = 0.0423; rank-biserial r = 0.23, 95% CI 0.02–0.45).

In addition, denture-related lesions were significantly more frequent in the de novo OSCC group (18.75%), whereas none were observed in the OSCC with OPMD group (*p* = 0.0117).

The overall distribution of histopathological grade did not significantly differ between groups (*p* = 0.206). Among patients with available histological grading, G1 carcinomas accounted for 25.9% of patients with OPMDs and 9.3% of those without OPMDs, whereas G2 carcinomas accounted for 55.6% and 65.1%, respectively.

**Figure 1:**
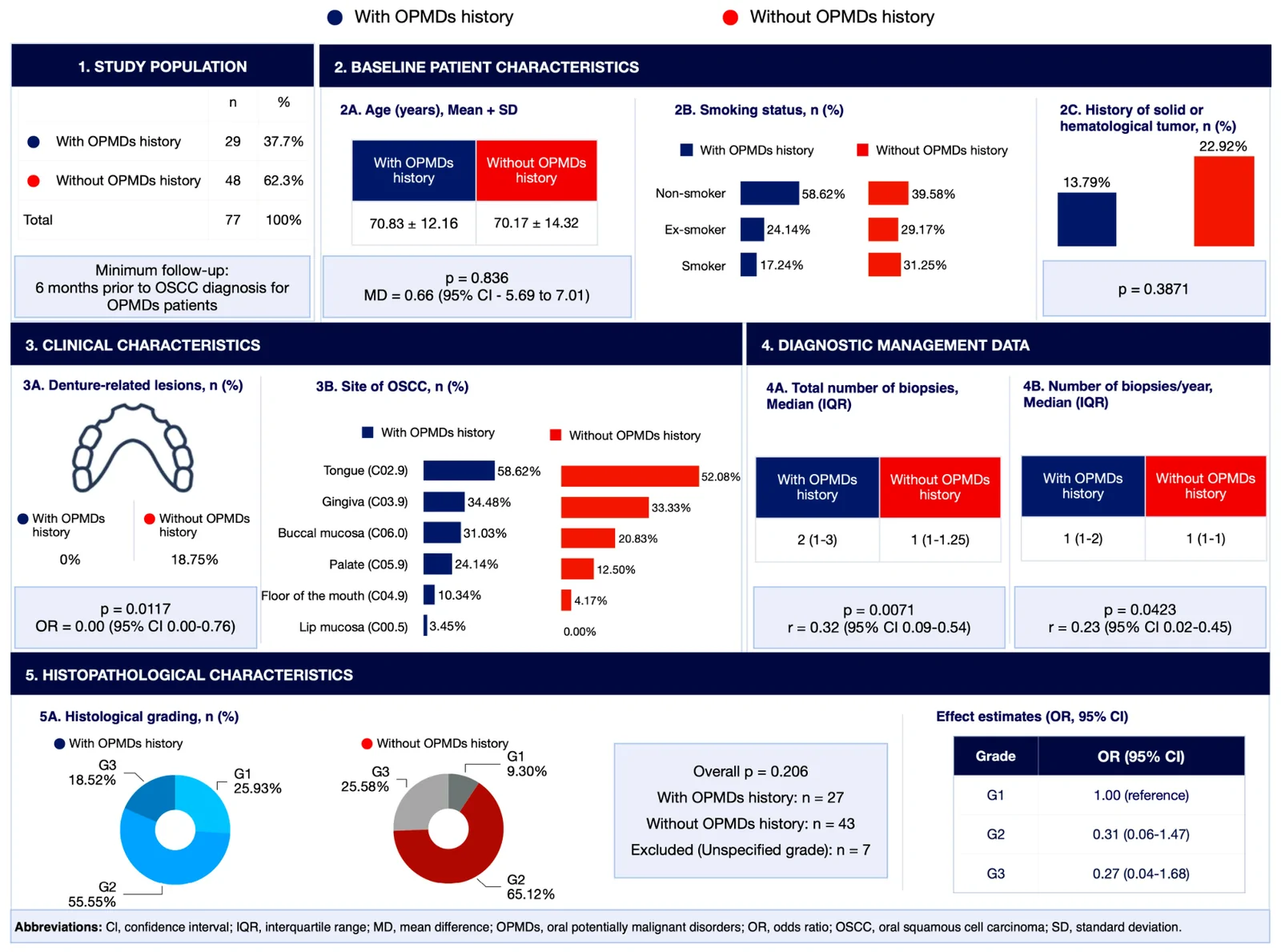
Graphical summary of the main characteristics of patients with oral squamous cell carcinoma (OSCC) according to the history of oral potentially malignant disorders (OPMDs).

Tumor anatomical distribution was overall similar between groups, with tongue localization remaining the predominant site in both cohorts. Although dorsal tongue involvement was observed exclusively among patients with OPMD history, the limited number of cases precluded meaningful statistical interpretation. A graphical summary of the main baseline patient characteristics, clinical and histopathological characteristics, and diagnostic management data according to the presence or absence of a history of OPMDs is presented in Figure 1.

A multivariable logistic regression analysis was performed to assess factors independently associated with a history of OPMDs among patients with OSCC (Table 3). The analysis included 70 patients with available histological grading (27 with OPMDs and 43 without OPMDs). After adjustment for age, sex, smoking status, and histological grade, none of the included variables showed a statistically significant independent association with a history of OPMDs. The adjusted OR was 0.84 per 10-year increase in age (95% CI 0.54–1.30; *p* = 0.431) and 1.86 for female versus male sex (95% CI 0.66–5.30; *p* = 0.243). Compared with non-smokers, the adjusted OR was 0.52 for former smokers (95% CI 0.15–1.87; *p* = 0.318) and 0.54 for current smokers (95% CI 0.12–2.37; *p* = 0.417). Compared with G1 tumors, the adjusted OR was 0.23 for G2 tumors (95% CI 0.05–1.05; *p* = 0.059) and 0.19 for G3 tumors (95% CI 0.03–1.20; *p* = 0.078). The overall effects of smoking status and histological grade were not statistically significant (*p* = 0.524 and *p* = 0.120, respectively).

## 4. Discussion

The present study aimed to characterize the clinicopathological profile of patients affected by OSCC, with particular focus on differences between tumors arising in association with OPMDs and those developing in the apparent absence of clinically detectable precursor lesions.

Overall, the population analyzed was predominantly composed of elderly patients, with a relatively balanced sex distribution and a predominance of moderately differentiated tumors, most frequently involving the tongue, gingiva and buccal mucosa. These findings are broadly consistent with the established epidemiological profile of OSCC, although recent literature has highlighted increasing variability in patient characteristics and clinical presentation [1,19,20].

The main finding of the present study is the absence of statistically significant differences between patients with and without OPMDs history with respect to traditional behavioral risk factors, including tobacco use, alcohol consumption, and HPV status. Tobacco and alcohol remain the most well-established contributors to oral carcinogenesis, with a well-documented synergistic effect [19,21], while HPV appears to play a limited and site-dependent role in OSCC, as also reflected by the low prevalence observed in our cohort [22,23].

Despite their recognized etiological relevance, the similar distribution of these variables between groups suggests that such factors alone may not fully explain the heterogeneity observed in clinical presentation. In particular, they do not appear to determine whether OSCC arises from a clinically detectable precursor lesion or develops in its apparent absence. This finding is consistent with Wang et al., who reported no significant differences in tobacco or alcohol exposure between OSCC patients with and without a history of OPMDs [24]. Fiedler et al. reported that OSCC in never-smokers/never-drinkers was more frequently associated with female sex, older age, and a history of OPMDs [25], suggesting that the relationship between traditional risk factors and OPMDs may vary across different patient populations.

In recent years, emerging epidemiological evidence has further reinforced this concept of heterogeneity. Several studies have reported a progressive increase in the incidence of OSCC, particularly oral tongue carcinoma, among younger patients and individuals without traditional risk factors such as heavy tobacco or alcohol use [26]. This trend has been observed across different geographic regions and populations, with some reports suggesting that early-onset OSCC may represent a partially distinct clinical entity characterized by different risk profiles and potentially different biological mechanisms [21,27]. Consistent with these observations, our findings support the hypothesis that oral carcinogenesis may involve multiple, partially independent pathways rather than a single linear progression model.

In our cohort, 37.66% of patients presented with a history of OPMDs, whereas 62.34% had no documented precursor lesions. This finding is in line with previous reports indicating that a substantial proportion of OSCC cases may arise without clinically identifiable premalignant conditions [28,29,30]. OPMDs constitute a heterogeneous group of disorders, exhibiting markedly variable malignant transformation rates, ranging from 1.4% to 49.5% over follow-up periods from 12 months to 20 years [8,28]. Therefore, the relatively lower proportion of OSCC cases with a documented OPMDs history observed in our study is not unexpected. These findings support the coexistence of different carcinogenic pathways, including tumors developing from recognized precursor lesions and carcinomas presenting apparently de novo. It should also be considered that some cases classified as de novo OSCC may actually represent lesions preceded by undiagnosed or undocumented OPMDs, which remained clinically unnoticed before the diagnosis of carcinoma [28,31].

Recent evidence has further strengthened the clinical relevance of this distinction. In a 2024 systematic review and meta-analysis, de novo OSCCs were more frequently associated with larger tumor size, more advanced stage, regional and distant metastases, and worse survival, whereas OSCCs arising in association with OPMDs were more often diagnosed at earlier stages [11]. Similarly, McCord et al. reported that OSCC associated with precursor lesions represented a minority of cases and was more frequently diagnosed at an earlier stage, with nearly half of cases identified as stage I. In contrast, tumors without documented precursor lesions more often presented at advanced stages and were associated with higher disease-specific mortality [32]. These findings highlight the potential benefit of recognizing and monitoring precursor lesions for earlier OSCC diagnosis, while also emphasizing the need to better characterize tumors developing without a documented OPMD history. The differences observed may reflect variations in timing of diagnosis, surveillance intensity, and potentially tumor differentiation patterns. In our cohort, the observed differences in histological grade distribution may also suggest differences in tumor differentiation or evolution between OPMD-associated and de novo OSCC. Confirmation of this hypothesis would require larger studies and the availability of TNM staging data.

Within this context, the concept of field cancerization may provide a useful interpretative framework. According to this model, genetically altered epithelial fields may extend beyond clinically visible lesions, predisposing to malignant transformation even in mucosa that appears macroscopically normal [12,33]. Although the retrospective nature of the present study does not allow direct investigation of molecular alterations, the occurrence of OSCC in the absence of detectable precursor lesions may be compatible with the presence of subclinical epithelial changes. This concept should be interpreted cautiously, as a plausible explanation rather than a demonstrated mechanism.

From a clinical perspective, the analysis of biopsy patterns offers additional insight into the differences observed between groups. Patients with OPMDs underwent a significantly higher number of biopsies, likely reflecting their inclusion in structured surveillance programs and closer clinical monitoring [30,34]. In contrast, in patients without clinically evident precursor lesions, the absence of suspicious mucosal alterations may reduce the likelihood of early diagnostic investigation. Our findings appear consistent with this interpretation: the higher biopsy burden observed in the OSCC with OPMD group may reflect more structured surveillance, whereas patients without documented precursor lesions may enter the diagnostic pathway only once the carcinoma has become clinically evident.

In this context, denture-related lesions may represent a further diagnostic challenge. Although chronic mechanical irritation caused by ill-fitting dentures has been proposed as a possible cofactor in oral carcinogenesis through persistent mucosal trauma and inflammation, these lesions are not currently classified as oral potentially malignant disorders and are generally regarded as reactive conditions without proven intrinsic malignant potential [28,35,36]. Their coexistence with OSCC may therefore contribute to delayed recognition, as they may clinically resemble benign traumatic alterations rather than established precursor lesions.

In addition to differences in biopsy patterns, well-differentiated carcinomas (G1) were more frequent among patients with OPMDs, whereas moderately differentiated tumors (G2) predominated in patients without documented precursor lesions. Although these findings should be interpreted cautiously due to the relatively limited sample size, they may suggest differences not only in timing of diagnosis and surveillance intensity, but also in tumor differentiation patterns or biological evolution between OPMD-associated and de novo OSCC.

This observation suggests that differences between OPMD-associated and de novo OSCC may be influenced not only by biological mechanisms but also by variability in clinical detectability and diagnostic pathways. Tumors arising in the context of OPMDs are inherently more likely to be identified during follow-up programs, potentially favoring earlier diagnosis and the detection of more differentiated lesions. Conversely, de novo carcinomas may remain clinically silent until later stages, although the possibility of partially distinct biological behavior cannot be excluded [37].

Overall, our results are consistent with recent literature describing increasing het-erogeneity in OSCC presentation, including cases occurring in patients without strong exposure to traditional risk factors or without a documented history of precursor lesions [19,20,23,38,39]. This evolving epidemiological landscape further supports the concept that OSCC is not a uniform disease but rather a spectrum of conditions with potentially different underlying mechanisms.

From a clinical standpoint, these findings suggest that surveillance of recognized OPMDs, although essential, cannot identify all patients who may develop OSCC. Clinical vigilance should therefore extend beyond patients presenting with classical behavioral risk factors or known precursor lesions. Dentists may have an important role in this context, as routine dental visits provide an opportunity for oral mucosal examination, recognition of suspicious lesions, patient counseling, and timely referral when indicated [40]. Greater awareness among the general population that OSCC may also occur in the absence of classical risk factors or recognized OPMDs may contribute to earlier recognition, and educational interventions may improve knowledge of oral cancer risk factors and signs [41].

Some limitations should be acknowledged. The retrospective design limits control over data completeness and introduces potential information bias. The relatively small sample size and monocentric setting may restrict the generalizability of the findings. The results should therefore be interpreted within the context of this specific Italian cohort. Differences in demographic characteristics, geographic setting, referral patterns, and exposure profiles may contribute to variations compared with other populations. The limited sample size also restricted the number of covariates included in the multivariable analysis and led to relatively wide confidence intervals for some estimates. Therefore, the lack of statistically significant independent associations should be interpreted with caution. Moreover, the possibility of underreporting or misclassification of OPMDs, particularly in patients referred from other institutions, may have influenced the results.

A further limitation of the present study is the lack of TNM staging and other oncological variables, including treatment and clinical outcomes. The inclusion of these parameters in future studies would allow a more comprehensive evaluation of tumor characteristics and strengthen the interpretation of the observed differences between patients with and without a history of OPMDs.

## 5. Conclusions

The present study highlights the heterogeneous nature of OSCC and supports the concept that tumors may develop in different clinical contexts, including carcinogenesis associated with OPMDs and OSCC occurring in the absence of a documented history of OPMDs (commonly referred to in the literature as de novo OSCC).

The absence of significant differences in the distribution of traditional risk factors between patients with and without OPMDs suggests that established etiological expo-sures, although central to OSCC development, may not fully explain the variability in clinical presentation. Rather, our findings indicate that differences between these groups may reflect a combination of factors related to clinical detectability and surveillance intensity, while potential differences in tumor differentiation patterns warrant further investigation.

These observations may have potential clinical implications, although these cannot be directly established from the present findings. Surveillance strategies focused on OPMDs remain essential; however, given that OSCC may also occur in patients without a documented history of precursor lesions, broader clinical vigilance could be considered. Further prospective, multicenter studies integrating clinical, histopathological, and molecular data will be necessary to better define the biological and clinical differences between OPMD-associated and de novo OSCC, and to refine risk stratification and early detection strategies.

## Figures and Tables

**Table 1:** Baseline patient characteristics, clinical, histopathological, and diagnostic management data of the study population.

Variables	Total (n = 77)
**Baseline patient characteristics**	
**Age:** average (SD)	70.42 (±13.47)
≤60	16 (20.78)
>60	61 (79.22)
**Gender:** n (%)	
Female	36 (46.75)
Male	41 (53.25)
**Tobacco status:** n (%)	
Non-smokers	36 (46.75)
Ex-smokers	21 (27.27)
Smokers	20 (25.97)
**Alcohol consumption status:** n (%)	
Drinkers	9 (11.69)
Ex-drinkers	2 (2.60)
Non-drinkers	66 (85.71)
**HPV history**: n (%)	
Yes	4 (5.19)
No	73 (94.81)
**Concomitant pathologies:** n (%)	
Yes	68 (88.31)
Coagulopathies	2 (2.94)
Cardiopathy	15 (22.06)
Arterial Hypertension	40 (58.82)
Respiratory diseases	2 (2.94)
Hypercholesterolemia	3 (4.41)
Thyroid disease	10 (14.71)
Bone disease	18 (26.47)
Diabetes mellitus	14 (20.59)
HCV	3 (4.41)
Fatty liver	1 (1.47)
No	9 (11.69)
**Solid or hematological Tumor history:** n (%)	
Yes	15 (19.48)
Skin	3 (20.00)
Breast	2 (13.33)
Oral	5 (33.33)
Blood	2 (13.33)
Oropharyngeal	1 (6.67)
Column	1 (6.67)
Thyroid	1 (6.67)
No	62 (80.52)
**Clinical characteristics**	
**Site of OSCC:** n (%)	
Tongue (C02.9)	42 (54.55)
Lateral border of the tongue (C02.1)	28 (66.67)
Ventral surface of the tongue (C02.2)	13 (30.95)
Dorsal surface of the tongue (C02.0)	3 (7.14)
Base of the tongue (C01.9)	3 (7.14)
Gingiva (C03.9)	26 (33.77)
Buccal mucosa (C06.0)	19 (24.68)
Palate (C05.9)	13 (16.88)
Floor of the mouth (C04.9)	5 (6.49)
Lip mucosa (C00.5)	1 (1.30)
**History of OPMDs:** n (%)	
Yes	29 (37.66)
Oral Lichen Planus	4 (13.79)
Oral leukoplakia	9 (31.03)
Proliferative Oral Leukoplakia	3 (10.34)
Oral Lichenoid Lesion	9 (31.03)
Erythroplakia	4 (13.79)
No	48 (62.34)
**Site of OPMDs lesions:** n (%)	
Nr. of involved sites: average (SD)	0.99 (±1.15)
Buccal mucosa	17 (22.08)
Lateral border of the tongue	16 (20.78)
Ventral surface of the tongue	15 (19.48)
Floor of the mouth	3 (3.90)
Hard and soft palate	8 (10.39)
Gingiva	17 (22.08)
**Denture-related lesions**	
Yes	9 (11.69)
No	68 (88.31)
**Oral candidiasis:** n (%)	
Yes	6 (7.79)
No	71 (92.21)
**Histopathological and diagnostic management data**	
**Histological grade** n (%)	
G1	11 (14.29)
G2	43 (55.84)
G3	16 (20.78)
Not specified	7 (9.09)
**Total nr. of biopsies:** average (SD)	2.1 (±2.0)
**Nr. of biopsies per year:** average (SD)	1.5 (±1.0)

Human papilloma virus (HPV); hepatitis C virus (HCV); oral squamous cell carcinoma (OSCC); oral potentially malignant disorders (OPMDs); standard deviation (SD).

**Table 2:** Comparison of clinicopathological characteristics between patients with and without oral potentially malignant disorders (OPMDs).

Variable	With OPMDs History (n = 29)	Without OPMDs History (n = 48)	*p*-Value	Effect Estimate [OR/MD/r] (95% CI)
**Baseline patient characteristics**				
**Age:** average (SD)	70.83 (±12.16)	70.17 (±14.32)	0.836	MD 0.66 (−5.69–7.01)
≤60 years	3 (10.34)	13 (27.08)	0.0915	1
>60 years	26 (89.66)	35 (72.92)		OR 3.18 (0.76–19.14)
**Sex:** n (%)				
Female	17 (58.62)	19 (39.58)	0.1569	OR 2.14 (0.77–6.16)
Male	12 (41.38)	29 (60.42)		1
**Tobacco status:** n (%)			0.2305	
Non-smokers	17 (58.62)	19 (39.58)		1
Ex-smokers	7 (24.14)	14 (29.17)		OR 0.56 (0.15–1.94)
Smokers	5 (17.24)	15 (31.25)		OR 0.38 (0.09–1.41)
**Alcohol consumption status:** n (%)			0.643	
Drinkers	4 (13.79)	5 (10.42)		OR 1.31 (0.24–6.72)
Ex-drinkers	0	2 (4.17)		OR 0.00 (0.00–9.19)
Non-drinkers	25 (86.21)	41 (85.42)		1
**HPV history:** n (%)				
Yes	2 (6.90)	2 (4.17)	0.6294	OR 1.69 (0.12–24.59)
No	27 (93.10)	46 (95.83)		1
**Concomitant pathologies:** n (%)				
Yes	28 (96.55)	40 (83.33)	0.1414	OR 5.50 (0.67–257.19)
Coagulopathies	0	2 (5.00)		
Cardiopathy	3 (10.71)	12 (30.00)		
Arterial hypertension	19 (67.86)	21 (52.50)		
Respiratory diseases	0	2 (5.00)		
Hypercholesterolemia	3 (10.71)	0		
Thyroid disease	4 (14.29)	6 (15.00)		
Bone disease	7 (25.00)	11 (27.50)		
Diabetes mellitus	5 (17.86)	9 (22.50)		
HCV	1 (3.57)	2 (5.00)		
Fatty liver	0	1 (2.50)		
No	1 (3.45)	8 (16.67)		1
**Solid or hematological tumor history:** n (%)				
Yes	4 (13.79)	11 (22.92)	0.3871	OR 0.54 (0.11–2.10)
Skin	0	3 (22.92)		
Breast	0	2 (18.18)		
Oral	3 (75.00)	2 (18.18)		
Blood	0	2 (18.18)		
Oropharyngeal	1 (25.00)	0		
Column	0	1 (9.09)		
Thyroid	0	1 (9.09)		
No	25 (86.21)	37 (77.08)		1
**Clinical characteristics**				
**Site of OSCC:** n (%)				
Tongue (C02.9)	17 (58.62)	25 (52.08)		
Lateral border of the tongue (C02.1)	8 (47.06)	20 (80.00)		
Ventral surface of the tongue (C02.2)	6 (35.29)	7 (28.00)		
Dorsal surface of the tongue (C02.0)	3 (17.65)	0		
Base of the tongue (C01.9)	1 (5.88)	2 (8.00)		
Gingiva (C03.9)	10 (34.48)	16 (33.33)		
Buccal mucosa (C06.0)	9 (31.03)	10 (20.83)		
Palate (C05.9)	7 (24.14)	6 (12.50)		
Floor of the mouth (C04.9)	3 (10.34)	2 (4.17)		
Lip mucosa (C00.5)	1 (3.45)	0		
**Denture-related lesions**				
Yes	0	9 (18.75)	0.0117	OR 0.00 (0.00–0.76)
No	29 (100)	39 (81.25)		1
**Oral candidiasis:** n (%)				
Yes	3 (10.34)	3 (6.25)	0.6668	OR 1.72 (0.21–13.79)
No	26 (89.66)	45 (93.75)		1
**Histopathological and diagnostic management data**				
**Histological grade:** n (%)			0.206	
G1	7 (25.93)	4 (9.30)		1
G2	15 (55.56)	28 (65.12)		OR 0.31 (0.06–1.47)
G3	5 (18.52)	11 (25.58)		OR 0.27 (0.04–1.68)
Not specified	2	5		Excluded from grade comparison
**Total number of biopsies:** median (IQR)	2 (1–3)	1 (1–1.25)	0.0071	r = 0.32 (0.09–0.54)
**Number of biopsies/year:** median (IQR)	1 (1–2)	1 (1–1)	0.0423	r = 0.23 (0.02–0.45)

Confidence interval (CI); hepatitis C virus (HCV); Human papilloma virus (HPV); interquartile range (IQR); mean difference (MD); oral potentially malignant disorders (OPMDs); oral squamous cell carcinoma (OSCC); odds ratio (OR); rank-biserial correlation (r); standard deviation (SD). Note: For the site of OSCC: the reported frequencies refer to all anatomical subsites involved by each tumor rather than exclusively to the primary tumor site.

**Table 3:** Multivariable logistic regression analysis of factors associated with a history of potentially malignant disorders (OPMDs) in patients with oral squamous cell carcinoma (OSCC).

Patient/Tumor Characteristic	Category	Adjusted OR	95% CI	*p*-Value	Overall *p*-Value
**Age**	Per 10-year increase	0.84	0.54–1.30	0.431	
**Sex**	Female vs. male	1.86	0.66–5.30	0.243	
**Smoking status**	Ex-smoker vs. non-smoker	0.52	0.15–1.87	0.318	0.524
	Current smoker vs. non-smoker	0.54	0.12–2.37	0.417	
**Histological grade**	G2 vs. G1	0.23	0.05–1.05	0.059	0.120
	G3 vs. G1	0.19	0.03–1.20	0.078	

Confidence interval (CI); oral potentially malignant disorders (OPMDs); oral squamous cell carcinoma (OSCC); odds ratio (OR). Note: The dependent variable was the presence of a history of OPMDs among patients with OSCC. The model included age, sex, smoking status, and histological grade. Patients with unspecified histological grade were excluded (n = 7); final model n = 70.

## Data Availability

The data presented in this study are available on request from the corresponding author. The data are not publicly available due to privacy and ethical restrictions.

## Abbreviations

The following abbreviations are used in this manuscript:

OSCC	Oral squamous cell carcinoma
OPMDs	Oral potentially malignant disorders
HPV	Human papillomavirus
HCV	Hepatitis C virus
SD	Standard deviation
G1	Well-differentiated carcinomas
G2	Moderately differentiated tumors
